# The Relationship between Jaundice and Weight Loss after Birth in Neonates Referring to the KOSAR Hospital, Qazvin-Iran: Short title: Jaundice and Weight Loss

**DOI:** 10.31661/gmj.v15i.3896

**Published:** 2026-01-30

**Authors:** MohammadReza Maleki, Fatemeh Samiee Rad, Masoomeh Hadi, Asghar Ghorbani

**Affiliations:** ^1^ Clinical Research Development Unit, Qods Hospital, Qazvin University of Medical Sciences, Qazvin, Iran; ^2^ Department of Pathology, Qazvin University of Medical Sciences, Qazvin, Iran; ^3^ Department of Pediatrics, Qazvin University of Medical Sciences, Qazvin, Iran; ^4^ Baharloo Hospital, Department of Pediatrics, Tehran University of Medical Sciences, Tehran, Iran

**Keywords:** Neonatal Hyperbilirubinemia, Neonatal Jaundice, Neonatal Weight Loss

## Abstract

**Background:**

This study aimed to determine the association between the incidence of neonatal jaundice and the weight loss of newborns in postnatal days.

**Materials and Methods:**

In this cross-sectional study, 200 infants with jaundice were examined. The required information was obtained from the medical records. In this study, newborns were divided into two groups based on hyperbilirubinemia. Bilirubin level was compared in two groups on the fourth day of referral. Effect of weight loss was determined on hyperbilirubinemia by logistic regression.

**Results:**

The study compared 137 healthy neonates with 53 hyperbilirubinemic neonates, finding significantly greater weight loss (0.25±0.18 kg vs. 0.09±0.11 kg, P0.001) and higher bilirubin levels (17.13±1.05 vs. 12.60±1.89 mg/dL, P0.001) in the latter group. Hyperbilirubinemic neonates had younger mothers (27.23±3.26 vs. 28.63±3.21 years, P=0.007), lower nulliparity (60.4% vs. 75.9%, P=0.033), and higher breastfeeding rates (96.2% vs. 18.6%, P0.001). Logistic regression confirmed weight loss as a strong predictor (aOR=2.14×106, P0.001), with ROC analysis showing high diagnostic accuracy (AUC=0.86-0.88). Optimal cutoffs were 0.175 kg (89% sensitivity) and 4.95% weight loss (91% sensitivity). Maternal and neonatal blood groups also influenced risk.

**Conclusion:**

Neonates at risk need to be more closely monitored for jaundice and weighting, thus, early detection and timely treatment can be done.

## Introduction

Neonatal jaundice is a common problem in infancy as it is seen in 60% of term infants and 80% of preterm infants during the first week [1]. Unconjugated bilirubin is the end product of heme catabolism in reticuloendothelial cells. High levels of indirect (unconjugated) bilirubin are potentially neurotoxic [1][2]. Jaundice can lead to many problems such as hospitalization of the baby, which leads to separation of the mother from the baby, economic costs, and encephalopathy due to hyperbilirubinemia, corn ectrosis and death [3][4][5]. The highest prevalence of severe hyperbilirubinemia has been reported in Asia [6]. Neonatal hyperbilirubinemia is the cause of one third of neonatal hospitalization in Iran [7]. Neonatal hyperbilirubinemia and its complications such as encephalopathy due to hyperbilirubinemia and corn ectrosis are a serious burden in developed and underdeveloped countries [8]. The most common diagnosis of neonatal jaundice is by measuring serum or cutaneous bilirubin and comparing it to standard curves. Clinical jaundice is seen in infants when serum bilirubin levels exceed 5 mg/dl [9].

Common and proven treatments for neonatal jaundice currently mainly include phototherapy and blood transfusions [10].

Some risk factors for unconjugated hyperbilirubinemia include polycythemia, infection, prematurity and diabetes mellitus, delayed meconium excretion, breastfeeding, and weight loss and dehydration [2]. In full-term and near-term infants who do not have any of the above risk factors and are exclusively breastfed, sometimes due to the mother's physical condition or insufficient training on breastfeeding due to insufficient calories and weight loss the baby develops jaundice [11][12].

Weight loss during infancy (both in infants who are exclusively breastfed and formula-fed) is commonly seen due to physiological dieresis [13][14]. Feeding exclusively on exclusively breastfed infants is low in the first few days after birth, so these infants lose more weight than formula-fed infants [15][16]. Weight loss of more than 3% before the start of weight gain in infants who are exclusively fed formula and more than 7-8% in infants who are exclusively breastfed is considered significant weight loss [17].

This difference in initial weight loss between breastfed and formula-fed infants is usually well tolerated, but can have serious clinical consequences. In infants formula-fed, weight loss of up to 10% (with severe weight loss) occurs rarely, but about 10% of infants who are exclusively breastfed and the result of vaginal delivery, and about 25% of infants who are exclusively breastfed and who are the result of cesarean delivery, could develop severe lose weight. This weight loss of 10% or more is associated with an increased risk of hypernatremia and hyperbilirubinemia [16][15].

The importance of preventing potential complications of hyperbilirubinemia and the relationship between neonatal jaundice and neonatal weight loss is the rate of weight loss of infants in the days after birth.

This study aimed to assess the relationship between jaundice and weight loss after birth in neonates.

## Materials and Methods

The present study is a cross-sectional study. The sample consisted of neonates with jaundice meeting the inclusion criteria and the parents of infants signed written consents to participate in the study. Inclusion criteria included healthy-infants over 35 weeks, weight measurement on the fourth day and its registration in KOSAR Hospital, and also have bilirubin on the days measured in the study, and birth weight over 2500 grams.

Exclusion criteria include neonates weighing less than 2,500 grams at birth, gestational age under 35 weeks, and any known risk factors for jaundice, including LBW prematurity, hemolysis evidence such as positive coombs, ratikolositoz, glucose-6-phosphate dehydrogenase deficiency (G6PD), hematoma, asphyxia infection, and major anomalies. The minimum sample size was 144 people. Taking into account the 20% loss, we included 200 subjects.

In this study, 200 healthy infants with jaundice who referred to the clinic of KOSAR Hospital in April and May 2017 were examined and the required information was prepared in a checklist that contains the studied variables including sex, gestational age, and type of delivery. Birth weight, weight at the time of referral, breastfeeding and formula, age of mother, degree of jaundice at the time of referral, adjuvant feeding with formula, absolute formula feeding with conscious consent were collected and entered in the file by a trained person. Neonatal jaundice was determined based on the definition of hyperbilirubinemia and according to the level of bilirubin at the time of referral. Bilirubin levels above 16 on the fourth day were compared.

After collecting the data, the data were entered into the Stata 17 (StataCorp) statistical software. The normality of data distribution was checked using the Kolmogorov-Smirnov test. Descriptive statistics compared groups using independent t-tests for continuous variables (reported as mean ± SD) and chi-square tests for categorical variables. Logistic regression assessed the association between weight loss and hyperbilirubinemia, first in an unadjusted model and then adjusted for maternal/neonatal covariates. The optimal weight loss cutoff was determined using the cutpt package (Liu method).

### Ethical Considerations

The research plan of this study was approved by the Research Ethics Committee of Qazvin University of Medical Sciences (ID: IR.QUMS.REC.1398.040) and the patient information was recorded in complete confidentiality. No additional costs were imposed on patients.

## Results

**Table:** Table1. Descriptive Characteristics of Neonates by Hyperbilirubinemia Status

**Variable**	**Healthy (n = 137)**	**Hyperbilirubinemia (n = 53)**	**Statistic (t/χ²)**	**P-value**
**Weight-related**				
Weight loss (kg), M (SD)	0.09 (0.11)	0.25 (0.18)	t = -7.12	<0.001
Birth weight (kg), M (SD)	3.23 (0.38)	3.27 (0.33)	t = -0.68	0.496
Second weight (kg), M (SD)	3.14 (0.37)	3.01 (0.32)	t = 2.36	0.019
**Bilirubin**				
Total bilirubin, M (SD)	12.60 (1.89)	17.13 (1.05)	t = -18.99	<0.001
**Demographics**				
Maternal age, M (SD)	28.63 (3.21)	27.23 (3.26)	t = 2.74	0.007
Gender (% male)	54.7%	54.7%	χ² = 0.00	0.999
**Obstetric**				
Gravidity, M (SD)	2.11 (0.88)	1.89 (0.87)	t = 1.58	0.116
Nulliparous (%)	75.9%	60.4%	χ² = 4.57	0.033
Delivery (% cesarean)	42.9%	49.1%	χ² = 0.61	0.434
**Feeding**				
Breastfeeding (%)	18.6%	96.2%	χ² = 96.42	<0.001
**Blood groups**				
Maternal Rh+ (%)	95.6%	90.6%	χ² = 1.72	0.190

**Table:** Table2. Unadjusted and Adjusted Logistic Regression for Hyperbilirubinemia

**Predictor**	**Unadjusted OR (95% CI)**	**Adjusted OR (95% CI)**	**P-value**
Absolute weight loss (kg)	4.60 × 10⁶ (3.63 × 10⁴, 5.82 × 10⁸)	2.14 × 10⁶ (7.88 × 10³, 5.81 × 10⁸)	< .001
Percent weight loss (%)	1.65 (1.41, 1.93)	1.59 (1.33, 1.90)	< .001
Male gender	—	2.13 (0.83, 5.48) / 1.98 (0.78, 5.03)*	.116 / .151
Maternal blood group	—	1.45 (1.00, 2.10) / 1.38 (0.96, 1.99)*	.049 / .081
Neonatal blood group	—	0.51 (0.34, 0.75) / 0.53 (0.36, 0.78)*	.001 / .001
Maternal Rh+	—	0.71 (0.11, 4.57) / 0.70 (0.11, 4.50)*	.717 / .703
Cesarean delivery	—	1.28 (0.49, 3.34) / 1.28 (0.49, 3.30)*	.609 / .615
Gestational age	—	0.95 (0.60, 1.51) / 1.01 (0.64, 1.59)*	.827 / .956
Maternal age	—	0.85 (0.71, 1.01) / 0.83 (0.70, 0.99)*	.062 / .040
Nulliparity	—	0.61 (0.19, 1.92) / 0.62 (0.20, 1.92)*	.398 / .405

The study included 137 neonates without hyperbilirubinemia and 53 neonates with hyperbilirubinemia. Neonates with hyperbilirubinemia (n=53) demonstrated significantly greater weight loss (0.25±0.18 kg vs. 0.09±0.11 kg, P<0.001) and higher bilirubin levels (17.13±1.05 vs. 12.60±1.89 mg/dL, P<0.001) compared to healthy controls (n=137). The hyperbilirubinemia group had younger mothers (27.23±3.26 vs. 28.63±3.21 years, P=0.007), lower nulliparity rates (60.4% vs. 75.9%, P=0.033), and near-universal breastfeeding (96.2% vs. 18.6%, P<0.001). No significant differences were observed in birth weight, gender distribution, gravidity, delivery mode, or maternal Rh status (all P>0.05). Logistic regression analyses demonstrated a significant association between neonatal weight loss and hyperbilirubinemia. In the unadjusted model, greater weight loss (measured both in absolute terms and as a percent change) markedly increased the odds of hyperbilirubinemia (absolute weight loss: OR=4.60 × 106, 95% CI [3.63 × 104, 5.82 × 108], P<.001; percent weight loss: OR=1.65, 95% CI [1.41, 1.93], p<.001). After adjusting for maternal and neonatal covariates (gender, maternal and neonatal blood group, Rh status, delivery mode, gestational age, maternal age, and nulliparity), both measures remained significant predictors (absolute weight loss: aOR=2.14 × 106, 95% CI [7.88 × 10³, 5.81 × 108], P<.001; percent weight loss: aOR=1.59, 95% CI [1.33, 1.90], P<.001). Maternal blood group (aOR=1.45, 95% CI [1.00, 2.10], P=.049) and neonatal blood group (aOR=0.51, 95% CI [0.34, 0.75], P=.001) were also significant predictors in the adjusted model. Note. OR=Odds ratio; CI=Confidence interval. Unadjusted models: n=190; Adjusted models: n=189. For absolute weight loss, ORs are interpreted per 1-unit increase (e.g., 1 kg); for percent weight loss, ORs are per 1% increase. Dual values reflect results from absolute and percent weight loss models, respectively.

**Figure-1:**
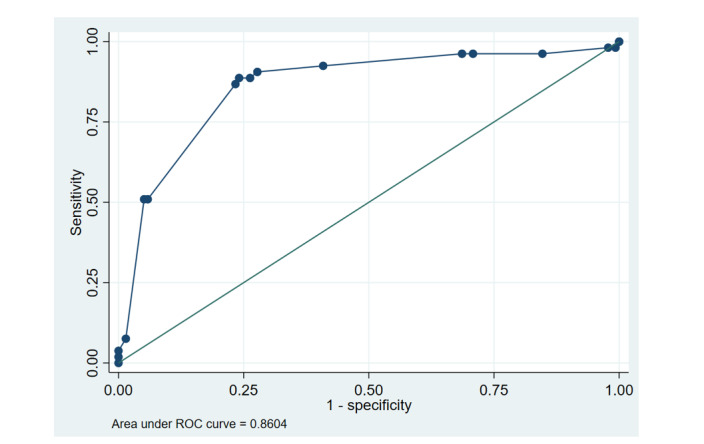
Figure 1. Receiver Operating Characteristic (ROC) Curve for Neonatal Weight Loss Predicting Hyperbilirubinemia

**Figure-2:**
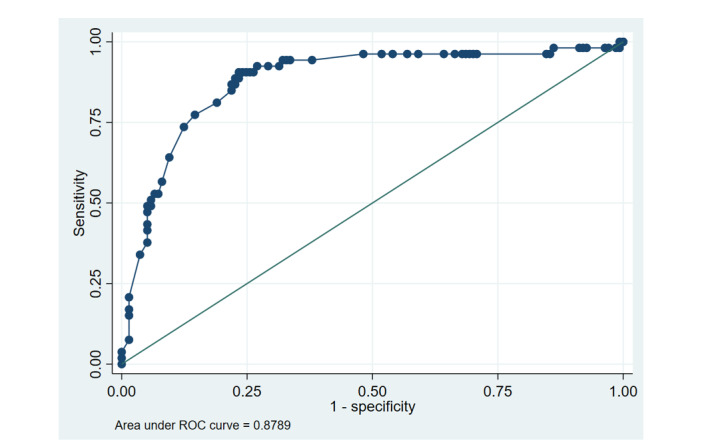
Figure 2. Receiver Operating Characteristic (ROC) Curve for percent of Neonatal Weight Loss Predicting Hyperbilirubinemia

Greater neonatal weight loss (both absolute and percent change) significantly predicted hyperbilirubinemia. The area under the ROC curve was 0.86 (95% CI [0.80, 0.92]) for absolute weight loss and 0.88 (95% CI [0.82, 0.94]) for percent weight loss (Figure-1 and -2 ). The optimal cutoff for absolute weight loss was 0.175 kg (89% sensitivity, 76% specificity), while the optimal percent weight loss cutoff was 4.95% (91% sensitivity, 77% specificity).

## Discussion

The present study investigated the association between neonatal hyperbilirubinemia and postpartum weight loss, along with other maternal and neonatal factors, in a cohort of 190 neonates. Our findings demonstrate a strong and statistically significant relationship between greater weight loss in neonates and the development of hyperbilirubinemia, even after adjusting for potential confounders. Neonates with hyperbilirubinemia exhibited nearly three times greater weight loss (0.25 ± 0.18 kg) compared to healthy controls (0.09 ± 0.11 kg, P<0.001), reinforcing the clinical relevance of early weight monitoring in predicting jaundice risk. Furthermore, logistic regression analysis revealed an exceptionally high odds ratio for hyperbilirubinemia per kilogram of weight loss (aOR=2.14 × 106), suggesting that even minimal weight fluctuations may serve as a critical early indicator.

Exclusive breastfeeding was nearly universal in the hyperbilirubinemia group (96.2% vs. 18.6%, P<0.001), aligning with previous studies that highlight the role of breastfeeding-associated jaundice due to caloric deprivation or insufficient milk intake. Our results support the hypothesis that exaggerated weight loss may reflect suboptimal feeding patterns, contributing to increased enterohepatic bilirubin circulation. Contrary to some prior research, we found no significant differences in birth weight, gestational age, or delivery mode between jaundiced and non-jaundiced neonates, suggesting that weight loss itself rather than baseline anthropometrics is the more critical predictor.

The ROC curve analysis identified a weight loss cutoff of 0.175 kg as optimal for hyperbilirubinemia prediction (89% sensitivity, 76% specificity), providing a clinically actionable threshold for early intervention. This finding is consistent with studies by Chang et al. and Salas et al., who similarly reported associations between postnatal weight loss and jaundice severity. However, our results contrast with Michel et al.’s earlier work, which found no significant correlation, possibly due to differences in sample size or measurement timing.

Similarly, Young et al. observed that infants losing ≥7% of birth weight had a higher incidence of hyperbilirubinemia, though their study also identified low gestational age as a contributing factor—a relationship not seen in our cohort (Yang et al., 2013). Our results, however, demonstrate an even stronger predictive value of absolute weight loss (≥0.175 kg) independent of gestational age, suggesting that early weight monitoring may be a more universal marker than previously recognized.

Our analysis aligns with Salas et al. (2009), who noted peak hyperbilirubinemia-related readmissions on the fourth postnatal day, coinciding with the timing of maximal weight loss in our study. However, unlike prior studies that emphasized low birth weight (<2.5 kg) as a risk factor, we found no significant difference in birth weight between jaundiced and non-jaundiced neonates. Instead, the critical predictor was postnatal weight loss magnitude, which was exacerbated in exclusively breastfed infants (96.2% of hyperbilirubinemia cases). This supports the hypothesis that breastfeeding-associated jaundice may stem from inadequate caloric intake rather than intrinsic neonatal factors. Discrepancies with studies like Huang et al. (2009), which reported heavier infants at higher jaundice risk likely reflect methodological differences, including our focus on absolute weight loss rather than percentile-based thresholds and variations in population demographics. Notably, Michel et al.’s null findings (1983) may have been limited by small sample size and assessment restricted to the first three days, underscoring the importance of extended monitoring as implemented in our protocol.

While neonatal-maternal factors may influence postnatal weight trends, our study found no significant association between hyperbilirubinemia and several key variables. Notably, gender distribution was balanced among jaundiced neonates (54.7% male vs. 54.7% female, P=0.999), contradicting some previous reports that suggested male predominance in hyperbilirubinemia (Ismailpour et al.; Mahmoudi et al.). This aligns with Huang et al.'s cohort study (2009), which similarly found no gender-based differences in jaundice risk.

Additionally, neither neonatal nor maternal Rh status showed significant correlation with hyperbilirubinemia in our analysis (P=0.190 for maternal Rh+), reinforcing findings from Saber et al. (2013) and Tavakolizadeh et al. (2018). These results suggest that while weight loss and breastfeeding patterns are strong predictors of neonatal jaundice, traditional factors like sex and Rh incompatibility may have limited predictive value in this context.

Some limitations of this study should be acknowledged. The cross-sectional nature of this study limits the ability to establish causality between neonatal jaundice and weight loss. It only provides a snapshot of data at one point in time, making it difficult to determine whether weight loss leads to jaundice or vice versa. The study may be prone to sampling bias, as the selection of participants might not accurately represent the broader neonatal population. This could affect the generalizability of the findings. The absence of follow-up data restricts insights into how neonatal jaundice and weight loss evolve over time or respond to interventions. Potential confounding factors, such as maternal health, feeding practices, socioeconomic status, and environmental influences, may not have been adequately accounted for, which could influence both jaundice and weight loss outcomes. The use of existing medical records as the primary data source may introduce inaccuracies or incomplete information that could affect the reliability of results. These limitations highlight the need for further research using longitudinal designs or cohort studies to better understand causality and address confounding factors comprehensively.

## Conclusion

Our updated findings underscore neonatal weight loss as a potent, independent predictor of hyperbilirubinemia, with exclusive breastfeeding and younger maternal age as contributing factors. Early identification of excessive weight loss, particularly beyond 0.175 kg—may facilitate timely interventions to mitigate jaundice severity, improving neonatal outcomes. Clinicians should consider integrating routine weight surveillance into postnatal care protocols, especially for exclusively breastfed infants.

## Conflict of Interest

There was no conflict of interest.
